# Supplementary material for: Machine learning for healthcare that matters: Reorienting from technical novelty to equitable impact
Source: PLOS Digit Health. 2024 Apr 15;3(4):e0000474. doi: 10.1371/journal.pdig.0000474 (PMC11018283; doi:10.1371/journal.pdig.0000474)
Supplement: Attachment — Submitted filename: MLHC that Matters_ Author Response Letter Final.docx [file pdig.0000474.s001.docx]

**To the Editor and Reviewers:**

We thank you for the thoughtful and constructive comments on our paper. It is not always the case in the academic publication process that comments are so detailed and well-informed. Our paper has significantly benefited from the reviews, we have updated the acknowledgement section of our paper to include thanks to our anonymous reviewers.

Our detailed responses are outlined below, and the specific changes are highlighted in red in the revised document.

***Reviewer #1: This is a very good article. My suggestions are few but important. All of them pertain to the last two sections of the framework.***

***1. Under "Evaluation: Re-Aligning Incentives", you advocate for an appreciation of audacious endeavors. What do you mean by audacious? On the one hand, you want to elevate the overlooked realm of implementation science (the "last-mile" work); on the other, you want to encourage bold ideas. Suggest you align both of these thoughts toward the real-world goal of equity, as demonstrated by your final 4 examples. Those examples (I think!) demonstrate what you mean by audacity. Just make that plain here. We want people to ask how they can create equity using the model -- which is very different than asking how they can manage the bias of the model. It's a question of goals rather than boundaries.***

This is an excellent point well-phrased, and I think it fully captures what we are striving to achieve with this work. The emphasis is upon ML as a means for producing equity, rather than the milder constraint of ML not worsening equity. This change has been made in the first paragraph of the section, borrowing some of your helpful warning.

***2. Same section, under "Recognizing Advances..." You mention that researchers compete for reviewer attention. You should be more explicit about grant money and industry funding. The political economy of science shapes the research, which biases the research (be audacious and say it!).***

We appreciate your encouragement in this regard. The section has been significantly expanded, with direct discussion of the role and impact of funding mechanisms, particularly in preventing projects from being carried forward throughout their entire lifecycle.

***3. Under "Moving on From Clinically Unimportant Metrics," you should add a recommendation about local testing and validation. It could fold into the clinical trials rec, though it's not exactly the same.***

We agree that while connected, this is an importantly distinct recommendation. The section has been updated to reflect this. We have also updated the recommendation regarding clinical trials for further clarity in this regard.

***4. Under "Encouraging Moonshots," lengthen this recommendation: "Allocate grant funding to ambitious projects with longer timelines, aiming at real-world clinical benchmarks and equitable outcomes."***

We have expanded this recommendation to reflect your comment.

***5. Under "Considering Human Processes," you ask three very good questions about how clinicians will interact with a model. I suggest you broaden the frame: what sort of habits are clinicians forming in relation to ML models? Then name specific risks, like automation bias and alarm fatigue (i.e. the habit of blindly following or blindly ignoring).***

This section has been expanded to reflect these comments.

***6. Under the same heading, in your recommendations, include: implement processes where clinicians can provide constant feedback. Establish who is accountable for the model, so that clinician feedback goes somewhere, and there are processes in place for continuous improvement (as well as triggers and backup plans for model downtime -- very important for patient safety).***

The section has been updated alongside these recommendations. We have also added a further recommendation (in line with your discussion of downtime procedures) addressing the issue of deskilling and the need to ensure that clinicians remain prepared to function in model-free circumstances.

***7. Under "Identifying System Limitations", you recommend that researchers should be rewarded for stating the limitations of a model. Please ground this. How should they be rewarded? By whom, and with what incentive?***

This is an excellent suggestion, and it underscores the importance of operationalizing our recommendations. We have updated with a pair of recommendations, highlighting the importance of peer review and publication in the academic domain, and the gatekeeping role of regulators in the domain of industry.

***8. Under "Building for Continued Impact," suggest you state that short-term rewards pose a risk to patients.***

Another great point - we have updated to reflect this, and included discussion of how healthcare processes can be altered in support of such projects in ways that are not easily reversed.

***9. In your recommendations for the same section, you recommend establishing institutional monitoring boards. "Boards" is too high-level. Suggest "teams and processes" or something like that.***

This is updated - we agree that the reference to “boards” can imply a distant administrative-level monitoring entity rather than something closer to the day-to-day of patient care needs.

***10. Under "Impact Challenges..." you provide four excellent examples. Suggest you stay with the equity focus on number 4, just by lengthening it: "...a >=90% physician satisfaction rate with answer usefulness across multiple health systems in diverse socioeconomic contexts."***

Thank you for this recommendation, which greatly enhances the parallelism of this section and clarity of our focus on equitable impact. It has been updated.

***Overall, a really sharp set of ideas. A companion piece should map them to the AI lifecycle to further ground how this would go in a stepwise fashion: problem definition, data engineering, model building, small-scale testing, deployment and monitoring.***

We look forward to the opportunity to spur further work in this domain, and to carry out some of this work ourselves!

***Reviewer #2: This landscape review highlights the disparity between the promise of machine learning for healthcare and the current state of the field, which emphasizes technical novelty over real world impact. The authors distinction between machine learning ON healthcare data and machine learning FOR healthcare is well articulated as is the framework proposed and recommendations suggested, which aim to achieve substantively meaningful and equitable clinical outcomes. Ultimately, the paper serves as a comprehensive critique of current state of the field but goes beyond simply pointing out shortcomings and concludes with impact challenges that refocus machine learning efforts within healthcare toward more meaningful societal impacts. This paper is an important contribution to the broader conversation around AI/ML and health equity and will be essential reading for all stakeholders in this space.***

***-	Fostering Diverse Dataset Creation***

***o	Consider citing Datasheets for datasets as an example of a framework for providing detailed descriptions of how training datasets were created and how they should be used.***

This is a great point, and certainly an oversight on our end not to cite. We have added.

***o	Do you have any recommendations for resource constrained institutions around developing datasets?***

We have expanded this section with some detailed discussion of current ongoing initiatives, as well as reference to the assistance beyond funding alone which is required to ensure that this data is accessible. We have also included additional citation to further work that has been recently published in this regard.

***-	Opening Data and Increasing Accessibility***

***o	What does it mean/look like to “ensure efforts toward open data are inclusive” across various settings?***

We have expanded this associated recommendation with particular reference to the importance of including a wide range of healthcare settings beyond the academic hospitals which typically dominate datasets in MLHC.

***-	Building Foundational Tools for MLHC Success***

***o	Consider also emphasizing tools and platforms for archiving and sharing files and data for rigor and reproducibility (i.e., Dataverse.org)***

Thank you for pointing us in the direction of this project - this is precisely the sort of collective open effort that we are hoping to emphasize. It has been added to the project.

***-	Understanding Social Determinants and Data Biases***

***o	Given the overarching goal of striving for equity, consider moving this section to the beginning above Fostering Diverse Dataset Creation.***

This is an excellent point, and helps to further emphasize the core focus of our work. The change has been made as suggested.

***o	Introduce the concept of data empathy, the context/story behind the data that give way to inherent biases in the data:*** [***https://www.liebertpub.com/doi/full/10.1089/big.2014.0026***](https://www.liebertpub.com/doi/full/10.1089/big.2014.0026)

Thank you for introducing this citation and concept to us - we agree that, particularly in healthcare, it is important not to lose the human stories underlying a data generation process. We have made changes to reflect this.

***-	Improving Diversity among Researchers***

***o	Provide some specific examples of how to incorporate diverse stakeholders into the ML design, development, and deployment processes.***

We have added further clarification for this section. Specifically, we have added discussion and citation regarding patient engagement practices which already exist in clinical trials. We have also included explicit discussion of the need to have those with a wide range of backgrounds and roles in the healthcare process itself (including patients, caregivers, and those such as homecare workers who are often not included in academic research development).

***o	Add recommendation about sustainable investments in capacity building at historically black and minority serving institutions, which disproportionately train underrepresented health and computer science trainees.***

This is an excellent point, and we have added an additional recommendation explicitly regarding the incorporation of investment into HBCUs and other minority-serving institutions in order to improve the training pipeline.

***-	Valuing Engineering Effort***

***o	Consider introducing/recommending a formal implementation/evaluation framework, like RE-AIM, to quantify engineering efforts and standardize reporting of “issues, dimensions, and steps in the design, dissemination, and implementation process that can either facilitate or impede success” of model deployments.***

We have added reference to the RE-AIM framework within the body of our text, and a paragraph discussing how its core goals are in line with our vision. In particular, there is a need to recognize that engineering effort to adapt to an existing complex healthcare context is core to, rather than ancillary to, an MLHC project. Establishing a similar formalization explicitly for MLHC would be an interesting further direction for this work, and it is one that we will consider in future work.

***-	Identifying System Limitations***

***o	Have you considered recommending “Model cards” as a tool to help characterize and ensure dissemination of limitations of models.***

In line with the recommendations around “Datasheets for Datasets”, we agree that “Model cards” are a foundational concept that deserves citation and discussion in our paper. We have added the citation and made changes reflecting this.

***-	Valuing Generalization On Par With Innovation***

***o	Introduce the Realist evaluation framework, which provides a formal methodology for probing “What works, for whom, in what respects, to what extent, in what contexts, and how?”***

This is a very useful methodology to summarize and respond to this question. Thank you for including it, and we have included brief discussion of it within this section.

***-	Other***

***o	Ensure to have periods as the end of every recommendation.***

This change has been made throughout the document.

***Journal Requirements:***

***1. Please review your reference list to ensure that it is complete and correct. If you have cited papers that have been retracted, please include the rationale for doing so in the manuscript text, or remove these references and replace them with relevant current references. Any changes to the reference list should be mentioned in the rebuttal letter that accompanies your revised manuscript. If you need to cite a retracted article, indicate the article's retracted status in the References list and also include a citation and full reference for the retraction notice.***

***2. Please send a completed 'Competing Interests' statement, including any COIs declared by your co-authors. If you have no competing interests to declare, please state "The authors have declared that no competing interests exist". Otherwise please declare all competing interests beginning with twhe statement "I have read the journal's policy and the authors of this manuscript have the following competing interests:"***

We have added the following to the text:

*The authors have declared that no competing interests exist.*

***3. Please ensure that Funding Information and Financial Disclosure Statement are matched.***

We have added the following to the text:

*Dr. I.B. is an employee of IBM Research. Dr. J.G. is a 2022 Robert Wood Johnson Foundation Harold Amos Medical Faculty Development Program and declares support from RSNA Health Disparities grant (#EIHD2204), Lacuna Fund (#67), Gordon and Betty Moore Foundation, and NIH (NIBIB) MIDRC grant under contracts 75N92020C00008 and 75N92020C00021. Dr. L.A.C. is is funded by the National Institute of Health through NIBIB R01 EB017205. Dr. T.N. is an employee of Microsoft Research. Dr. U.S is partially funded by the Israeli Science Foundation grant no. 1950/19. Dr. M.vdS. is supported by the Office of Naval Research (ONR). Dr. K.W. is an Independent Researcher.*

*The funders had no role in study design, data collection and analysis, decision to publish, or preparation of the manuscript.*

***4. In the Funding Information you indicated that no funding was received. Please revise the Funding Information field to reflect funding received.***

We have updated this in the online system.

***5. Please provide separate figure files in .tif or .eps format only and remove any figures embedded in your manuscript file. Please also ensure that all files are under our size limit of 10MB.***

This has been done in the final document.

**MISCELLANEOUS:**

We have updated Dr. Kiri L. Wagstaff’s name to include her middle initial at her request.

WIth thanks for your consideration,

Liam G. McCoy; Division of Neurology, Department of Medicine, University of Alberta

Aparna Balagopalan; Department of Electrical Engineering and Computer Science, Massachusetts Institute of Technology

*On behalf of all authors.*
